# Mechanism of OH*-Modified 4H-SiC Surface with Scratches Based on ReaxFF MD Simulation

**DOI:** 10.3390/mi16020184

**Published:** 2025-02-03

**Authors:** Dongxiao Yan, Hui Huang, Mingpu Xue, Nian Duan

**Affiliations:** 1 Institute of Manufacturing Engineering, Huaqiao University, Xiamen 361021, China; 2 State Key Laboratory of High-Performance Tools, Huaqiao University, Xiamen 361021, China; 3 College of Mechanical Engineering and Automation, Huaqiao University, Xiamen 361021, China

**Keywords:** plasma, ReaxFF MD, 4H-SiC, OH*

## Abstract

OH* generated through plasma catalysis offers several advantages, including a long survival time, high modification efficiency, and environmental friendliness. Consequently, a plasma-assisted polishing technology has rapidly developed. Previous studies exploring the interaction mechanism between OH* and 4H-SiC have often assumed flat surfaces. However, in the surface modification experiments on 4H-SiC, the actual surface morphology was not flat but contained numerous scratches. Therefore, this study investigated the interaction mechanism of OH* on an uneven surface using reactive force field molecular dynamics (ReaxFF MD) simulations. The results show that in the low-speed OH* modification process, the adsorption effect leads to a thicker modified layer at higher locations than at lower locations. The resulting modified layer can be removed by soft abrasive mechanical polishing to achieve surface flatness, but there will be a modified layer on the surface, which needs to be modified and polished several times. In contrast, during high-speed OH* modification, high-speed particle bombardment causes more Si-O bonds to diffuse into the scratches, resulting in the formation of a flat bonding layer with surface planarization achieved after a single polishing step. The interaction mechanism of OH* with the uneven surface at different speeds, as obtained through ReaxFF MD, provides a theoretical foundation for subsequent polishing experiments.

## 1. Introduction

Currently, among more than 200 crystal types of silicon carbide (SiC), the most used include 3C-SiC, 4H-SiC, and 6H-SiC. Among these materials, 4H-SiC is notable, owing to its high electron mobility, superior breakdown field strength, enhanced thermal conductivity, and better lattice matching [1,2,3], which enable heterogeneous integration with other materials, such as gallium nitride (GaN). These outstanding characteristics make 4H-SiC ideal for a wide range of applications, including new energy vehicles, power transmission systems, and wireless communication technologies [4,5]. In these industrial applications, the 4H-SiC substrate has extremely high-quality requirements. Its total thickness deviation should not exceed 3 μm, the curvature and warpage should be within 25 μm, and its surface roughness must not surpass 0.5 nm. Moreover, the surface must be free of scratches and without any subsurface damage. These all fall into the realm of high-precision machining demands.

Although 4H-SiC offers numerous advantages, its high hardness and chemical inertness pose significant challenges to its processing. The processing of 4H-SiC wafers typically involves several stages, including slicing, grinding, and polishing [6,7,8]. As the final step in wafer processing, polishing is crucial for determining the quality of subsequent SiC device fabrication. Currently, chemical-mechanical polishing (CMP) is widely used to polish 4H-SiC wafers [9,10]. This process achieves surface flattening through a combination of the chemical reactions of chemical agents (KOH and KMnO₄) and mechanical removal by soft abrasives. However, the use of chemical agents complicates waste treatment and contributes to environmental pollution [11]. In contrast to chemical reagents such as strong acids and bases, OH*, the second most potent oxidizer (oxidation potential of 2.80 V) in nature after fluoride, reacts more readily with 4H-SiC, reducing its surface hardness. Consequently, OH* is widely [12,13,14] used in polishing 4H-SiC.

The main methods for polishing 4H-SiC using catalytically produced OH* include plasma-assisted polishing [15,16,17], photocatalysis [18,19], and the Fenton reaction [20,21,22]. In plasma-assisted polishing, OH* is generated by the ionization and excitation of water molecules by high-energy electrons. The 4H-SiC surface after OH* modification is softened, with its hardness lower than that of the original 4H-SiC. The modified layer is mechanically removed using a soft abrasive, resulting in a flattened surface. The OH* generated via plasma catalysis exists in gaseous form, which offers several advantages over the OH* produced in solution by photocatalysis or the Fenton reaction, such as longer survival time, higher modification efficiency, and a more environmentally friendly catalytic process, consequently leading to the rapid development of plasma-assisted polishing techniques. Yamamura et al. [23] employed a capacitively coupled discharge (CCP) to ionize helium (He) and water-mixed gas, which produced a plasma jet containing OH* that assisted in polishing 4H-SiC, achieving a surface roughness (Ra) value of 0.423 nm. Similarly, Deng et al. [24] obtained a smooth surface with a p-v of 1.889 nm and a root mean square (rms) value of 0.280 nm using plasma-catalyzed OH* polishing. Deng et al. [25] conducted a plasma modification rate experiment and compared it with the thermal oxidation rate at 1100 °C. The plasma modification rate was six times higher than that of thermal oxidation. The above experimental research on plasma-assisted polishing shows that plasma can modify SiC at a relatively high rate. In a previous study, we explored the interaction mechanism between OH* and 4H-SiC at the atomic scale using ReaxFF MD simulations [26]. During the OH* modification of 4H-SiC, C atoms leave the surface as gaseous compounds, leaving Si atoms to form Si-O bonds with O atoms, which reduces the surface hardness. After reducing the surface hardness, it can be mechanically polished by soft abrasive particles to achieve surface flatness. Free radicals produced by plasma-induced ionization were used to replace strong acids and bases in the process of chemical-mechanical polishing.

However, in our previous studies, the surface was assumed to be flat. Considering surfaces often contain numerous scratches, exploring the interaction mechanism between OH* and a scratched surface instead of a flat surface would offer more valuable insights into subsequent polishing experiments. In summary, this study investigated the mechanism of OH* interaction with an uneven 4H-SiC surface based on ReaxFF MD simulations, providing theoretical guidance for future polishing experiments.

## 2. Simulation

### 2.1. Deposition Velocity

Plasma is a collection of electrically neutral substances comprising electrons, ions, and neutral particles [27,28]. OH* is a neutral particle; its velocity is not influenced by the electric field. Typically, their velocities are determined by thermal motion and follow a Maxwell distribution function. Plasma can be categorized into high- and low-temperature types based on temperature [29,30,31]. Low-temperature plasmas can be further subdivided into hot (<10,000 K), warm (<3000 K), and cold plasmas (<1000 K). Different types of plasma exhibit varying temperatures. For instance, warm plasma is frequently employed in fields such as surface modification and film deposition, while cold plasma is often utilized in biomedicine, environmental protection, and surface modification owing to its low-temperature characteristics [32,33].

**Figure 1:**
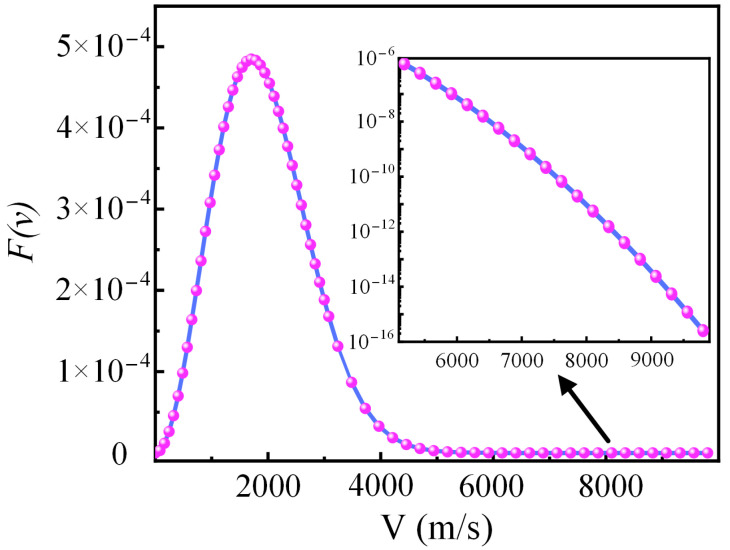
Thermal motion distribution function of OH* at 3000 K.

By taking the warm plasma temperature of 3000 K as an example, the OH* velocity distribution function is shown in Figure 1. The x axis is the velocity distribution of OH*, and the y axis is the probability of occurrence of pair velocity. When the OH* velocity exceeded the peak value, the probability of OH* appearing at the corresponding velocity decreased with increasing velocity. Currently, atmospheric pressure discharge is the most common form of plasma used in polishing processes. Under atmospheric pressure, the number density of OH* in plasma typically ranges from 10^15^ to 10^17^ m^−3^ [34]. The OH* velocity distribution under these conditions ranged from 0 to 10,000 m/s. The kinetic energies of OH* at 10 and 100 m/s are relatively small, and the number of particles at these velocities is low; thus, they are negligible. Although the number of high-speed OH* particles was small, their kinetic energy was significant, and their contributions could not be disregarded. Therefore, the deposition simulations were primarily conducted at three different speeds: 1000 m/s, 5500 m/s, and 10,000 m/s.

### 2.2. Simulation Model

**Figure 2:**
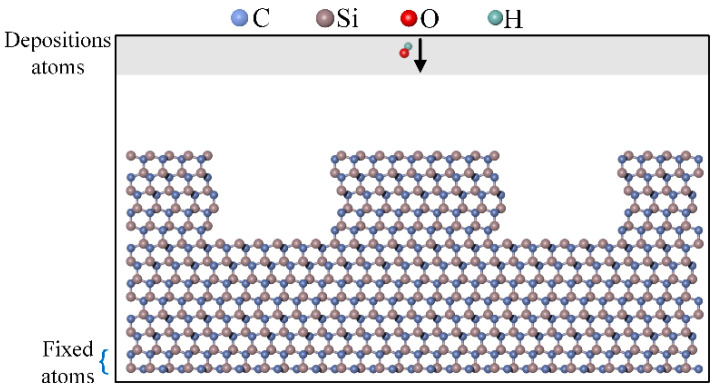
Schematic diagram of the simulation model.

A straight-edge scratch morphology was established on the surface via the pairwise removal of Si-C atoms. The simulation model is shown in Figure 2. The simulation process was described from three perspectives: bottom, side, and top. The model consisted of a deposition layer and a fixed layer. Periodic boundary conditions were applied in the X-and Y-directions, whereas the Z-direction was constrained by non-periodic and fixed boundary conditions. The elements involved in the potential function [35,36] are C, H, O, and Si. The size of the 4H-SiC model is 80 × 21 × 43 Å, containing 4956 particles. OH* radicals were randomly generated in the deposition layer and vertically deposited onto the 4H-SiC surface. The time step for the simulation was 0.25 fs, and 600,000 steps were executed. During plasma irradiation of 4H-SiC, in addition to the chemical interaction between OH* and the surface, inert gas particles physically bombard the surface, generating extremely high instantaneous temperatures. Therefore, setting the temperature of 4H-SiC to 2000 K simulates the effect of inert gas particles in generating high instantaneous temperatures and accelerates surface chemical reactions, allowing for better observation of the OH*-modified surface phenomena.

## 3. Simulation Result

### 3.1. The Deposition Results of OH* at Different Times When Deposited at 1000 m/s

**Figure 3:**
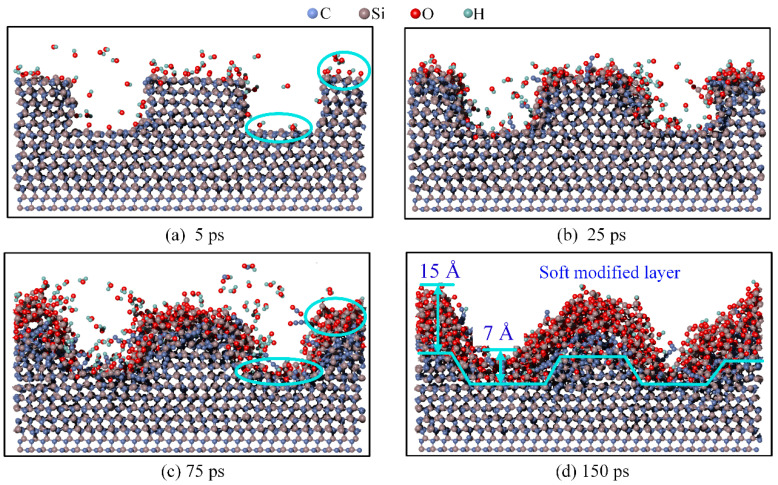
Results at different times of deposition at 1000 m/s. (**a**) 5 ps; (**b**) 25 ps; (**c**) 75 ps; (**d**) 150 ps.

The deposition results at different times and at a speed of 1000 m/s are shown in Figure 3. Specifically, for deposition times of 5 ps, 25 ps, 75 ps, and 150 ps, the corresponding numbers of running steps are 20,000, 100,000, 300,000, and 600,000, respectively.

At 5 ps, as shown in Figure 3a, the number of OH* deposited on the top surface is significantly greater than that deposited on the bottom surface. At 25 ps, as shown in Figure 3b, owing to the higher number of OH* groups at the top, the 4H-SiC surface at the top began to exhibit clear reactions, forming more Si-O bonds. In contrast, the number of OH* groups at the bottom was small, and only a few OH* groups reacted with the 4H-SiC surface. At 75 ps, as shown in Figure 3c, the top surface begins reacting with the third layer of 4H-SiC, whereas the bottom surface continues reacting with only the first layer. As the modified reaction progressed, the original straight-edge scratch began to show a slight bevel, and the Si-O bonds gradually diffused into the scratch.

At 150 ps, as shown in Figure 3d, the thickness of the modified layer at the top is 15 Å, while the thickness at the bottom is 7 Å. The modified layer at the top was thicker than that at the bottom. Owing to particle bombardment, the original straight-edged side of the scratch becomes inclined. The step thickness at the interface between the modified layer and the 4H-SiC surface was significantly reduced compared to that of the initial surface, facilitating subsequent mechanical removal.

### 3.2. The Deposition Results of OH* at Different Time When Deposited at 5500 m/s

**Figure 4:**
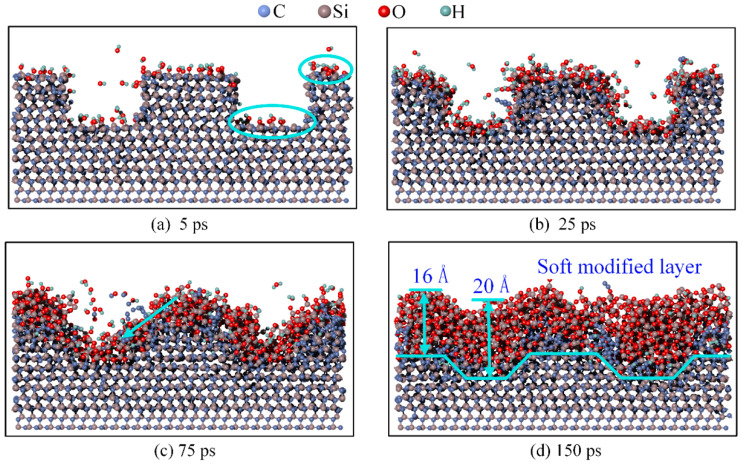
Results at different times of deposition at 5500 m/s. (**a**) 5 ps; (**b**) 25 ps; (**c**) 75 ps; (**d**) 150 ps.

The deposition results at different times and at a speed of 5500 m/s are shown in Figure 4.

At 5 ps, as shown in Figure 4a, the number of OH* molecules deposited at the bottom surface significantly increased compared to Figure 3a. At 25 ps, as shown in Figure 4b, the higher deposition speed resulted in more OH* entering the scratch. The number of OH* groups at the top and bottom was similar, resulting in a more uniform surface modification. At 75 ps, as shown in Figure 4c, with an increase in the deposition speed and kinetic energy, the number of Si-O bonds bombarded into the scratch also increased. Compared with Figure 3d, the surface exhibited an inclined edge 75 ps earlier.

At 150 ps, as shown in Figure 4d, the thickness of the modified layer within the scratch is 20 Å, while the thickness on both sides is 16 Å. The thickness of the modified layer at the bottom was greater than that on the sides, which is the opposite of the result obtained at 1000 m/s. During deposition, the modified layer within the scratch consists of the layer generated by the reaction between the deposited OH* and 4H-SiC and the layer formed by diffusion. Only the modified layer generated by the reaction between OH* and the 4H-SiC surface was present on both sides. Consequently, the modified layer within the scratch was thicker than that on the sides. However, at 1000 m/s, the step thickness between the modified layer and 4H-SiC remained similar to that of the initial surface and was significantly reduced.

### 3.3. The Deposition Results of OH* at Different Times When Deposited at 10,000 m/s

**Figure 5:**
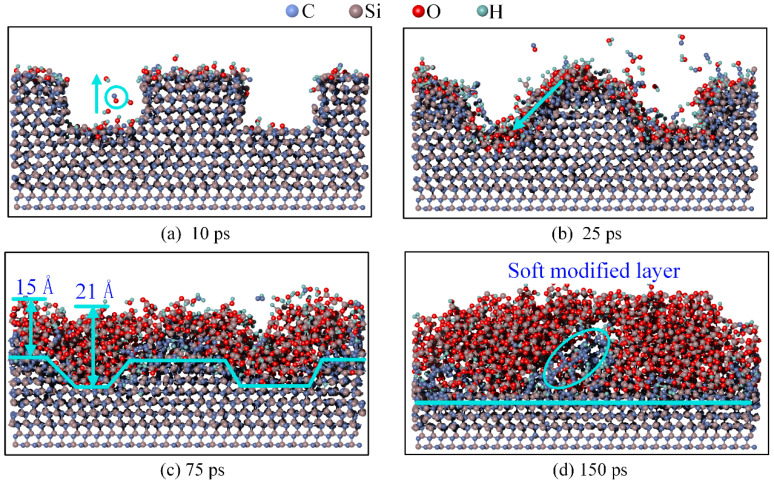
Results at different times of deposition at 10,000 m/s. (**a**) 10 ps; (**b**) 25 ps; (**c**) 75 ps; (**d**) 150 ps.

The deposition results at different times and at a speed of 10,000 m/s are shown in Figure 5.

At 5 ps, as shown in Figure 5a, owing to the increased velocity, no OH* offset was observed along the X and Y axes during the simulation. The higher velocity accelerates the reaction rate, and C atoms leave the surface as gaseous compounds. At 25 ps, as shown in Figure 5b, the deposition result is similar to that in Figure 4c; the straight-edge scratch becomes inclined, and the Si-O bonds diffuse into the scratch. At 75 ps, as shown in Figure 5c, the deposition result is similar to that shown in Figure 4d, with the thickness of the modified layer within the scratch being higher than that on both sides. The thickness of the modified layer within the scratch is 21 Å, while that on the sides is 15 Å.

At 150 ps, as shown in Figure 5d, a flat bonding layer formed between the modified layer and 4H-SiC. At 75 ps, the thickness of the modified layer on the scratch was greater than that on either side of the scratch. This increased thickness within the scratch led to a lower modification rate compared with that on the sides. Owing to the difference in the reaction rates, a dynamic equilibrium will eventually be reached between the two, and a flat bonding layer will form between the modified layer and 4H-SiC. During the simulation, C atoms aggregated to form carbon chains. This carbon chain formation phenomenon was also observed by Sun et al. [37] during oxidation of 3C-SiC.

### 3.4. Results of Different Speeds at an Inclined-Edge Scratch

**Figure 6:**
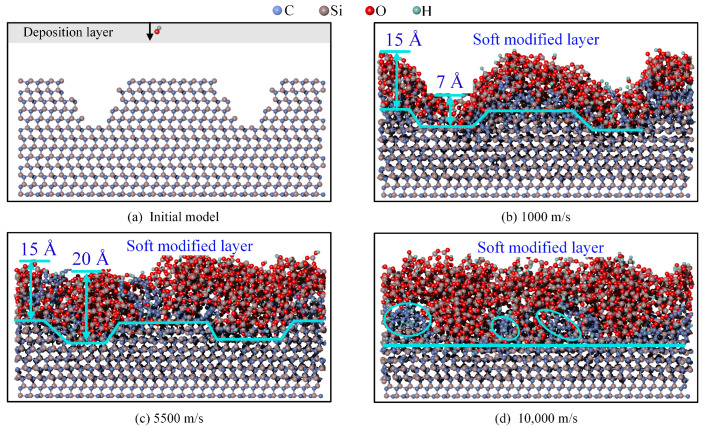
Result for an inclined-edge scratch. (**a**) Initial model; (**b**) 1000 m/s; (**c**) 5500 m/s; (**d**) 10,000 m/s

In addition to straight-edge scratches, inclined-edge scratches may occur on the surface. Therefore, a simulation model for inclined-edge scratches was developed, as shown in Figure 6a. Simulations were performed at three different speeds, with the results shown in Figure 6b–d. The deposition result at 1000 m/s, as shown in Figure 6b, is similar to that of the scenario with a straight-edge scratch. The modified layer formed on both sides was thicker than that formed in the scratch, with some Si-O bonds diffusing into the scratch. When the deposition speed is increased to 5500 m/s, the result in Figure 6c indicates that the situation is similar to the straight-edge scratch case, with a thicker modified layer on the scratch than on the sides. At a deposition speed of 10,000 m/s, as shown in Figure 6d, a relatively flat modified layer was formed above the 4H-SiC, similar to the straight-edge scratch results. However, in the inclined-edge scratch, the number of C atoms increased, whereas the rate at which C atoms left the surface remained constant, leading to a higher number of carbon chains. Overall, the surface modification by OH* was not significantly affected by scratch morphology.

## 4. Discussion

**Figure 7:**
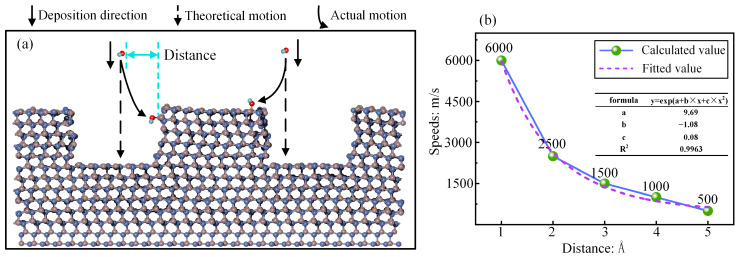
The details of the adsorption process: (**a**) the movement trajectory of OH*; (**b**) speed threshold at different distances.

During low-speed deposition, the OH* approach of 4H-SiC was influenced by chemical adsorption forces. Consequently, the OH* originally directed toward the bottom of the scratch alters its trajectory and is adsorbed on the sides or the top. The deposition process is shown in Figure 7a. Owing to the larger number of OH* groups on both sides, the thickness of the modified layer formed on the sides was greater than that in the scratch. As the deposition speed increased, the influence of the adsorption forces on the deposition direction of OH* decreased, as shown in Figure 7b. The minimum speed threshold required for OH* to reach the bottom is shown for different distances of OH* from the sides. However, as the speed increased, the bombardment effect of the particles accelerated the modification reaction and the diffusion rate of the Si-O bonds into the scratch, causing the modified layer in the scratch to become thicker than that on the sides during the process. At this point, the large thickness of the modified layer in the scratch limited the modification rate. As the reaction progressed, a flat modified layer formed.

**Figure 8:**
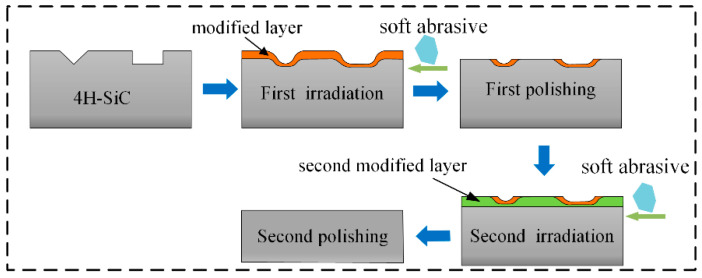
Polishing mechanism at low speed.

Based on the above simulations and the varying morphologies of the modified layer obtained at different deposition speeds, two distinct polishing mechanisms are proposed: high-speed OH* and low-speed OH*. The first mechanism is low-speed OH*, with the interaction mechanism between the low-speed OH* and 4H-SiC illustrated in Figure 8. The irradiated surfaces are also irregular. Owing to the influence of the adsorption forces, the thickness of the modified layer formed on the sides was greater than that formed in the scratch. Through nano-indentation tests, we previously demonstrated [26] that the hardness of the modified layer was lower than that of unmodified 4H-SiC. After plasma modification, a surface without subsurface damage can be obtained by the mechanical removal of soft abrasive particles without strong acid or alkali reagents; however, a small amount of the modified layer remains on the surface. At this point, a second plasma irradiation step was applied. The modified layer formed in the scratch during the first plasma exposure limited the modification rate, causing the modification rate in the scratch to be lower than that on the sides. This difference in the modification rate led to the eventual formation of a flat modified layer across the surface. Subsequent mechanical removal using a soft abrasive resulted in a flat surface without any residual modified layer. The number of cycles required depended on the thickness of each modification and the scratch depth.

**Figure 9:**
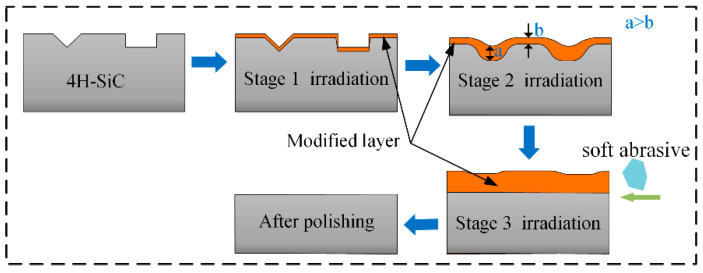
Polishing mechanism at high speed.

The polishing mechanism between the high-speed OH* and the surface is illustrated in Figure 9. This interaction is relatively complex and can be divided into three stages. In the first stage, owing to the high-speed movement of OH*, the influence of the adsorption force decreased during deposition, resulting in the formation of a uniform modification layer on the surface. In the second stage, the reaction continues, and the modified layer in the scratch is composed of OH* deposited through its reaction with 4H-SiC and Si-O bonds that diffuse into the scratch due to particle bombardment. Consequently, the modified layer at the bottom was thicker than that at the top. In the third stage, the increased thickness of the modified layer at the bottom limits the modification rate. Therefore, the modification rate at the top is higher than that at the bottom. As the reaction progressed, the modification rates at both the top and bottom eventually reached a dynamic equilibrium, leading to the formation of a flat modified layer across the surface. After the flat modified layer was formed, it was removed by soft abrasive polishing, resulting in a smooth, flattened surface.

## 5. Conclusions

In this paper, the mechanism of OH* deposition at different speeds on 4H-SiC surfaces with varying morphologies using ReaxFF MD is analyzed. The following conclusions were drawn:

1. During low-speed deposition, owing to surface adsorption, the modified layer formed at the top was larger than that formed at the bottom. Surface flattening can be achieved by modifying the polishing cycles.

2. During high-speed deposition, an increase in speed reduces surface adsorption; however, high-speed deposition accelerates the diffusion of Si-O bonds into the scratch, causing a difference in the modification rates between the bottom and top. Finally, a flat bonding layer was formed on the surface; this layer was flattened using a single polishing step.

3. When the OH* modified the 4H-SiC surface, two different scratch morphologies could obtain a similar, modified layer, indicating that the surface morphology had little effect on the modification result.

## Data Availability

Data will be made available on request.
